# Primary Sjogren’s Syndrome Associated With Treatment-Resistant Obsessive–Compulsive Disorder

**DOI:** 10.3389/fpsyt.2017.00124

**Published:** 2017-07-11

**Authors:** Lawrence T. C. Ong, Gary Galambos, David A. Brown

**Affiliations:** ^1^Centre for Immunology, Westmead Institute for Medical Research, University of Sydney, Sydney, NSW, Australia; ^2^St Vincent’s Private Hospital, Sydney, NSW, Australia; ^3^Westmead Hospital, Sydney, NSW, Australia

**Keywords:** Sjogren’s syndrome, obsessive–compulsive disorder, major depressive disorder, treatment resistance, autoimmune serology

## Abstract

There is an increasing awareness that autoimmune diseases can present with neuropsychiatric manifestations. We present the case of a 17-year-old female requiring psychiatric hospitalization for obsessive–compulsive disorder and major depressive disorder with mixed affective features, who was subsequently diagnosed with primary Sjogren’s syndrome. Treatment with potent immunosuppression resulted in remission of psychiatric illness. Due to a lack of awareness and/or the lack of specific biomarkers, clinicians may not associate psychiatric symptoms with autoimmune disease, including primary Sjogren’s syndrome. This case demonstrates that Sjogren’s syndrome may be a causative or aggravating factor in mental disorders and that autoimmune diseases should be carefully considered in the differential diagnosis of psychiatric illness especially in cases of concurrent physical symptomatology and severity or treatment resistance of psychiatric disease.

Although there is increasing awareness that a subset of psychiatric presentations may result from underlying autoimmune disease, the evidence for an association between autoimmune disease and obsessive–compulsive disorder (OCD) specifically, appears modest, and is perhaps strongest in conditions such as rheumatic fever (1). Here, we present a case of OCD, which appears to be associated with underlying autoimmune disease, most likely primary Sjogren’s syndrome (pSS) and discuss the need for a high degree of suspicion in diagnosis and timely management of such presentations.

Written, informed consent was obtained for the publication of the following case report. A 17-year-old female was admitted to a private psychiatric unit with features consistent with severe OCD. The patient suffered from recurrent obsessions regarding contamination, hoarding and symmetry, and compulsions including hand washing, showering, cleaning, and checking. Her symptoms had caused significant distress and interference with academic and social functioning, to the extent that she had become isolated from her peers and was unable to attend school. There was no drug use or diagnosed medical illnesses to account for her symptoms. Therefore, her symptomatology satisfied DSM-IV criteria for diagnosis of OCD. A score of 36 out of 40 on the Yale-Brown Obsessive–Compulsiveness score (YBOCS) indicated that symptoms were extreme in severity.

In addition to satisfying DSM-IV criteria for OCD, she also satisfied criteria for major depressive disorder, with melancholic features—in particular psychomotor agitation. She had been referred from a local general hospital after presenting there with suicidal ideation. A psychologist in the community had been managing her with psychotherapy for a number of months but her mental state had continued to deteriorate. She had been commenced on amitriptyline 10 mg daily by her family physician 2 weeks prior to admission, which was replaced fluoxetine 20 mg daily 1 week prior to admission.

There was no history of previously diagnosed mental disorder, although frequent nocturnal awakenings had been a problem for most of her life. Obsessive–compulsive symptoms were noted during primary school years, including checking the alignment of objects on a wall and checking that power points had been switched off. As a child, the patient demonstrated age-appropriate developmental milestones. The patient’s academic performance was considered to be superior throughout her school career. This was, however, interposed with a history of bullying and later depressive symptoms following a relationship breakup.

Her past medical history included facial lacerations from a dog attack in early childhood, subsequently requiring plastic surgery, tonsillectomy, adenoidectomy, and grommet insertion. There was a family history of anorexia nervosa, systemic lupus erythematosus (without cerebral lupus), but not OCD.

The patient was hospitalized and fluoxetine was increased to 80 mg daily (for her OCD and depressive features). In addition, quetiapine 350 mg daily (for her agitation) and prazosin 2 mg daily (for her nightmares) were commenced, in conjunction with group therapy and individual psychotherapy with her psychiatrist. There was a modest partial improvement in her condition.

Interestingly, the patient also complained of fleeting, non-specific and non-reproducible sensations in her head and upper body, fatigue and intermittent perceptual disturbances, both simple auditory hallucinations in the form of tinnitus (such as a cicada) and fleeting simple visual hallucinations (such as “a man dressed in black walking past”), which she had experienced every few weeks over the year previous to her current admission. There was an awareness by the inpatient treating team of the potential for autoimmune disease to present with psychiatric symptoms in young adults, so she was referred to an immunologist who was considered a member of the multidisciplinary team. An assessment for evidence of underlying autoimmune disease was conducted.

Autoimmune serology showed a positive antinuclear antibody with a speckled pattern (ANA titer >1:640). Extractable nuclear antigen antibody testing was also positive for SSA (Ro60 and Ro52) and SSB. Serum electrophoresis showed a large polyclonal increase in gammaglobulins (22 g/L, normal 4–12 g/L) and rheumatoid factor was also elevated at 64 IU/mL. Other markers associated with systemic lupus erythematosus such as anti Sm, anti dsDNA antibodies were not elevated and complement levels were normal. Thyroid function tests, ASOT and DNAseB were normal, while syphilis serology was negative and vitamin B12 and folate levels were replete.

Further history was sought regarding clinical manifestations of Sjogren’s syndrome (SS). There was a history of sicca a year prior to admission, which appeared to resolve spontaneously. Around the time of admission, a purpuric rash was noted on the legs, which was not biopsied, but demonstrated features suggestive of cutaneous vasculitis. There were no associated arthralgias or other extra-articular manifestations of SS.

Brain imaging in the form of a cerebral single photon emission computed tomography did not show evidence of cerebral hypoperfusion. Magnetic resonance imaging (MRI) showed no intracranial structural abnormalities in the cerebral hemispheres, cerebellum, brain stem, or intracranial arteries. Magnetic resonance spectroscopy, however, showed reduction in *N*-acetylaspartate (NAA) levels in both hippocampi, greater on the right side, which was suggestive of neuronal dysfunction. There were no abnormal myo-inositol or choline levels seen and no lipid or lactate peak with normal FA values throughout the cerebral hemispheres.

Further evidence of cerebral inflammation was sought through cerebrospinal fluid (CSF) analysis, which showed a pleocytosis with a white cell count of 10 × 10^6^/L (predominantly mononuclear cells), red cell count of 2 × 10^6^/L, elevated protein (429 mg/L, normal <400 mg/L), neopterin (24 nmol/L, normal <13 nmol/L), CSF IgG (111 mg/L, normal 1–40 mg/L), CSF IgG to albumin ratio (55%, normal 1–14%) and the presence of CSF-restricted oligoclonal bands. Antibodies to neuronal cell surface antigens including voltage gated potassium channel antibodies (LGI and CASPR2) and *N*-methyl-d-aspartate receptor antibodies were not detected in the CSF.

Three months following the initial admission, the patient developed a rash typical of urticarial vasculitis. She was commenced on prednisolone 25 mg daily and methotrexate 10 mg weekly. This was escalated to 15 mg weekly, 2 weeks subsequently. On review 2 months later, there had been a significant improvement in residual symptoms of OCD and mood disorder. The vasculitic rash was still present, but had improved in extent and frequency.

Five months following the initial admission, a worsening of depressive symptoms and suicidal ideation resulted in a further but brief hospitalization. Pulsed methylprednisolone was administered intravenously over 3 days with a significant improvement in mental state after her second dose. Psychotropic medications had been maintained continuously from the initial hospitalization, with the exception of a slight increase in quetiapine to 400 mg daily during the admission for elevated anxiety and agitation.

Given the initial improvement with immunomodulatory therapies, consideration was given to further intensifying treatment. Thus, 7 months following her initial admission, a course of plasmapheresis was given with five exchanges over a 10-day period. This was followed by intravenous immunoglobulin at 0.4 g/kg/day for 5 days and monthly maintenance doses at 0.4 g/kg on each occasion. Psychotropic medications were maintained continuously from the initial hospitalization.

A marked improvement was noted following plasmapheresis, such that her OCD symptomatology had completely resolved. There were, however, residual depressive symptoms noted in the week prior to each intravenous immunoglobulin infusion. The vasculitic rash had resolved completely. Ten months following her initial admission, the patient had recovered to the extent that she was able to enroll in a course in commercial bakery and participate in part time employment. Psychiatric treatment had remained essentially unchanged following the second psychiatric admission.

## Discussion

Primary Sjogren’s syndrome is a chronic autoimmune disease, which predominantly manifests with symptoms of sicca, but can have many and varied extraglandular manifestations including arthralgias, peripheral nervous system (PNS), and central nervous system (CNS) manifestations. Although recognized as potentially complicating the illness, PNS and CNS symptoms are not incorporated in the currently accepted classification criteria (2), which instead rely on a combination of sicca symptoms, glandular dysfunction, serology, and histopathology to classify patients with pSS.

The suspicion of associated autoimmune disease in this case was triggered by non-specific physical and neurological symptoms as well as the severity and treatment refractory nature of the psychiatric illness. Subsequent history, examination, and serology was most consistent with SS, and significant derangements in CSF markers (pleocytosis, elevated neopterin, CSF-restricted oligoclonal bands) indicated the presence of a neuroinflammatory process.

The association between pSS and OCD was underscored by the response to immunomodulatory therapy. Initial treatment with SSRI, atypical antipsychotic, and alpha antagonist resulted in mild improvement in symptoms within 2–4 weeks. Initial immunomodulatory therapy in the form of plaquenil, methotrexate, and prednisone was commenced approximately 2.5 months later due to persistent, partially treated symptoms. This resulted in rapid improvement as would be expected with glucocorticoid treatment of autoimmune disease. Prednisone was decreased 2 weeks later due to mood related side effects; however, by this time, checking behaviors had already ceased. Probably as a result of this and the slow onset of action of methotrexate, the patient was readmitted 1 month subsequently with increased agitation, decline in mood and suicidal ideation, at which point she was treated with IV methylprednisolone. Plasmapheresis was commenced approximately 2 months after this admission. This produced the most definitive change in mental state, with very rapid improvements within days of treatment, in the context of psychotropic medication being unchanged. On the basis of this, there appears to have been an association between pSS and OCD in this patient, although we cannot be conclusive about its nature. It is possible that pSS directly gave rise to OCD symptomatology, however, the patient’s premorbid vulnerability and isolated obsessive–compulsive characteristics might suggest that underlying OCD was exacerbated pSS.

Although the details presented in this case report do not completely fulfill the classification criteria for pSS, we contend that the serological evidence and response to immunomodulatory therapy makes the diagnosis of pSS most likely. The lack of lupus associated serological markers makes a diagnosis of SLE less likely, and pediatric autoimmune neuropsychiatric disorder associated with group A streptococci is also unlikely due to the age of onset, lack of history of streptococcal infections, and normal ASOT and DNAseB. Nevertheless, this cannot be completely excluded. Another weakness of this case report is the absence of objective markers of psychiatric improvement, which could have been addressed with serial measurements on the YBOCS or other psychiatric evaluation tool.

The neuroimaging is also worthy of discussion in this case. CNS disease in pSS can either be focal or diffuse, and although MRI is able to detect focal disease, it may not always detect diffuse disease (3) as in our patient. The MR spectroscopy results are also interesting as hippocampal changes are not typical of functional neuroimaging studies, which implicate the cortico-striatal-thalamo-cortical circuit in the pathophysiology of OCD (4). Despite this, hippocampal MR spectroscopic abnormalities have been previously found in OCD patients (5) and it is possible that the changes seen in our patient reflect this. Alternatively, low hippocampal NAA levels have been found previously in depressed adolescents (6). The hippocampal abnormalities may have correlated clinically with subjective cognitive dysfunction (the patient was unable to complete her higher school leaving examinations), although this was not formally tested. MR spectroscopic changes have also been found in patients with pSS, most notably decreased NAA levels or NAA/Cr ratios in the subcortical frontal and basal ganglia white matter (7). Although the subject of this report did not demonstrate these changes, a large proportion of patients in the aforementioned study also failed to show these MRS changes. More studies are required to characterize the range of MR spectroscopic abnormalities in pSS and their associated disease manifestations.

The published literature reports significant variability in estimates of CNS involvement in pSS with manifestations such as spinal cord dysfunction, seizures, migraines, and movement disorders occurring in 0–60% of patients (3). Estimates of the prevalence of neuropsychiatric disease in pSS are even more varied, ranging from 7 to 100% of individuals with pSS (3). This probably relates to difficulties in attributing psychiatric disorders to pSS as they may (1) arise concurrently, but independently of pSS, (2) arise in response to pSS, or (3) arise directly as a result of the pSS disease process. Although more research and clarification is required regarding the true incidence of neuropsychiatric disease due to pSS, data suggest that it may not be as rare as first thought, and may be missed due to subclinical disease, change in acuity over time or a low index of suspicion.

Attributing psychiatric disease to pSS is also complicated by variability in clinical presentation. Women with pSS rated themselves higher on symptoms of somatization, depression, anxiety, and paranoid ideation than healthy controls (8). Other studies have reported cognitive impairment (9), psychosis (9, 10), mood disorders (11–13), anxiety disorders (12, 13), somatization disorders (12), and dissociative states (12) or a combination of these in pSS patients. Despite this, very few published studies comprehensively phenotype the neuropsychiatric manifestations of pSS, disease course, and response to treatment. This information might better allow clinicians to differentiate pSS-related neuropsychiatric disease from primary psychiatric disease.

One final point of discussion is the recognition that CNS injury secondary to pSS may lead to permanent and irreversible damage. Some of the suggested mechanisms include direct CNS infiltration by mononuclear cells (14), vascular injury due to anti-Ro antibodies (15) and small vessel vasculitis (16). Because of this, it is particularly important that CNS manifestations of pSS be recognized and treated promptly.

In conclusion, we have presented the case of a patient with OCD and major depressive disorder that was associated with primary Sjogren’s syndrome. Although the attribution of mental disorder to pSS may be difficult, the potential for persistent, undertreated psychiatric morbidity, or permanent neurological damage should prompt timely referral of suspected cases to an immunologist or rheumatologist skilled in this field for further assessment and consideration of immunomodulatory therapy.

## Ethics Statement

Informed patient consent was obtained for purposes of publishing this case report.

## Author Contributions

The manuscript was prepared by LO and revised by GG and DB, both of whom also cared for the patient who was the subject of this case report.

## Conflict of Interest Statement

The authors declare that the research was conducted in the absence of any commercial or financial relationships that could be construed as a potential conflict of interest.
